# Pathogenic oral bacteria in hospitalised patients with dysphagia: The silent epidemic

**DOI:** 10.4102/sajcd.v68i1.798

**Published:** 2021-07-30

**Authors:** Merryl J. Weimers, Mershen Pillay

**Affiliations:** 1 Department of Speech Pathology and Audiology, Faculty of Health Sciences, University of KwaZulu-Natal, Durban, South Africa; 2 Speech and Language Therapy, Massey University, Auckland, New Zealand; 3 Department of Health Professions, Manchester Metropolitan University, Manchester, United Kingdom

**Keywords:** dysphagia, oral hygiene, aspiration pneumonia, oral bacteria, hospitalised patients

## Abstract

**Background:**

Aspiration pneumonia is a serious and fatal complication of dysphagia, secondary to the ingestion of bacteria-laden secretions. However, no studies have documented the oral hygiene features present in patients who present with dysphagia.

**Objectives:**

The purpose of this study was to describe the oral hygiene problems of adults admitted to a sub-acute rehabilitation hospital and who presented with dysphagia.

**Methods:**

A descriptive, cross-sectional survey was conducted, during which 40 participants – 57.5% (*n* = 23) male and 42.5% (*n* = 17) female – underwent a clinical swallow evaluation using the Mann Assessment of Swallowing Ability (MASA) augmented with cervical auscultation (CA) and pulse oximetry (PO), an oral hygiene assessment using an adapted version of the Oral Health Assessment Tool (OHAT), followed by microbiology laboratory analysis of buccal swab samples to detect bacteria not considered part of the normal oral flora.

**Results:**

Results indicated that poor oral hygiene status was a common feature amongst all participants who presented with dysphagia. The most prevalent oral hygiene issues were related to abnormalities concerning saliva (60%), oral cleanliness (82.5%), the tongue (80%) and the use of dentures (71.4%). A high prevalence, 62.5% (*n* = 25), of opportunistic bacteria was found. The most commonly occurring bacteria groups were: (1) *Candida albicans* (47.5%) and (2) respiratory pathogens (37.5%) such as *Klebsiella pneumoniae* and *Staphylococcus aureus*.

**Conclusion:**

Persons with dysphagia have poor oral hygiene which creates favourable environments for bacteria to flourish and increases the prevalence of pathogenic oral bacteria associated with the development of aspiration pneumonia. The management of oral health issues for persons with dysphagia should receive greater attention during hospitalisation.

## Introduction

The oral cavity of hospitalised and bedridden patients is often a reservoir for opportunistic respiratory pathogens (Boaden et al., 2017; Tada & Miura, 2012), and can thus serve as a source of infection for the lungs if aspirated. There is a significant correlation between the presence of pathogenic bacteria in the oral cavity and the occurrence of respiratory diseases, such as pneumonia, especially in people who present with dysphagia (swallowing impairment) (Fernández & Clavè, 2013; Konradsen, Trosborg, Christensen, & Pedersen, 2012; Simpelaere, Nufflen, Vanderwegen, Wouters, & De Bodt, 2016; Sørensen et al., 2013; Yoon & Steele, 2012). Swallowing is a complex neuromuscular activity that consists of oral, pharyngeal and oesophageal phases, and involves the coordinated function of many muscles (Hiramatsu, Kataoka, Osaki, & Hagino, 2015). Physiological deficits in any of the swallowing phases result in dysphagia (González-Fernández & Daniels, 2008) and may affect swallow efficacy (deficits with mechanical clearance) and/or safety of the swallow (subsequent aspirations). Impaired safety of the swallow not only increases the risk for aspiration and the uncontrolled introduction of pathogenic pathogens into the lower respiratory tract (Fernández & Clavè, 2013), but has also been associated with the presence of gram-negative bacteria; dysphagia is thus an important risk factor associated with the development of aspiration pneumonia (AP).

The incidence of pneumonia caused by aspiration of pathogenic bacteria in patients who present with dysphagia, increases both the mortality and the need for acute care hospitalisation (Sørensen et al., 2013), which in turn can impact on oral hygiene (Danckert, Plummer, & Williams, 2016; Neto, De Paula Ramos, Sant’ana, & Passanezi, 2011; Tada & Hanada, 2010). This is partly because of the fact that oral infections often remain asymptomatic and may still result in bacteraemia despite an absence of overt symptoms (Rautemaa, Lauhio, Cullinan, & Seymour, 2007). Oral care practices are often an overlooked component in the care of hospitalised patients (Chipps et al., 2014) as poor oral hygiene is not a highly visible condition, and related concerns, such as gingivitis and dental plague require a closer inspection of the mouth (Yoon & Steele, 2012). Furthermore, potentially pathogenic bacteria are frequently carried in the oral cavity and hospitalisation appears to create conditions that favour oropharyngeal colonisation (Boaden et al., 2017). The problem, however, is that dysphagia is often a symptom of multiple diseases and disorders, such as neurological disorders, cerebrovascular and neurodegenerative diseases that often require hospitalisation. Moreover, these diseases and disorders are often accompanied by impaired communication making dysphagia and its repercussions such as oral bacterial colonisation a highly invisible disorder and a silent epidemic. Unless we increase our knowledge and strengthen our understanding of the risk posed by poor oral hygiene to individuals with dysphagia, opportunistic respiratory bacteria originating in the mouth have the potential to become the silent killer of vulnerable hospitalised patients who present with dysphagia.

## Methods

### Study design and setting

A descriptive, cross-sectional study was conducted over an 8-week period, at a sub-acute neuro-rehabilitation hospital, in the private sector, in the Eastern Cape province of South Africa.

### Participants

The study included 40 participants, 57.5% (*n* = 23) male and 42.5% (*n* = 17) female, aged 20–99. Non-probability, convenience sampling was used to select participants for the clinical swallow evaluation (CSE). As per the admission protocol of the research site, all new patients undergo an initial screening of swallow function on the day of admission, by the resident speech therapists. Thus, potential participants were approached based on their dysphagia status as established on the day of admission. Patients were excluded if they did not meet inclusion criteria, for example, if they were minors, if they were on a non-dysphagic diet or if consent was not provided. Purposive sampling was used to recruit participants for the oral health assessment, as only those who were confirmed as having dysphagia, following the completion of the CSE underwent the oral health assessment.

The demographic, clinical diagnosis and nutritional status of participants included in the study are shown in Table 1.

**TABLE 1:** Demographic details of participants who presented with dysphagia (*N* = 40).

Variable	Characteristic	Number	%
Gender	Male	23	57.5
	Female	17	42.5
Age in years	20–59	13	32.5
	60–99	27	67.5
Diagnosis	Stroke	17	42.5
	Traumatic brain injury	2	5.0
	Anoxic brain damage	1	2.5
	Haemorrhage	6	15.0
	Cancer	3	7.5
	Meningitis	2	5.0
	Neurological conditions	3	7.5
	Other conditions	5	15.5
	No conditions	3	7.5
Enteral feeds (non-oral)	Percutaneous endoscopic gastrostomy	13	32.5
	Nasogastric tube	4	10.0
Dysphagia diet (texture modification)	Puree	13	32.5
	Minced and moist	4	10.0
	Soft and bite sized	6	15.0
Level of consciousness	No response	8	20.0
	Difficult to rouse	1	2.5
	Fluctuates	6	15.0
	Alert	25	62.5

## Data collection

Data were collected during two stages: (1) a CSE and (2) an oral health assessment. The oral health assessment consisted of two components, namely visual inspection of the oral cavity and microbiology laboratory testing of oral swab samples.

### Clinical swallow evaluation

During the CSE, the swallow function of each of the 40 participants recruited was assessed using the Mann Assessment of Swallowing Ability (MASA). The MASA is a bedside swallow assessment which measures 24 different skills pertaining to the oro-motor and sensory components of swallowing, to determine a patient’s swallow ability, in terms of efficacy and safety (Mann, 2002). Furthermore, the MASA contains several discriminate items reflecting cognitive status, level of alertness and cooperation. As a result, it could effectively be used on participants who were unconscious or presenting with cognitive deficits (Kwon et al., 2019). The MASA score is measured using a 5–10 point rating scale, with 200 being the highest possible score. Scores were used to characterise the clinical features of dysphagia, as well as provide an estimate of the risk of aspiration.

The MASA assessments were augmented with cervical auscultation (CA) and pulse oximetry (PO). Cervical auscultation was used to listen to the sounds of swallowing using a paediatric stethoscope to differentiate between normal and impaired swallow sounds (Cichero & Murdoch, 2006; Groher & Crary, 2010) and PO was used to obtain participant oxygen saturation (SpO^2^) levels during swallowing. For this study, PO was used to confirm the association between swallowing abnormalities and oxygen desaturation, seen as the difference ≥ 3% between baseline SpO^2^ and SpO^2^ after the swallow. Whilst on their own, CA and PO provide contestable data about swallowing and/or associated respiratory function, they were included as adjuncts to the MASA to increase the sensitivity and specificity of the assessment methods.

### Oral health assessment

The oral health assessment was used to characterise the oral hygiene status of participants as well as the degree of oral bacterial colonisation.

#### Oral Health Assessment Tool

To ensure that the most accurate and reliable findings regarding each participant’s oral hygiene were obtained, a qualified oral hygienist used an adapted version of the Oral Health Assessment Tool (OHAT) (Chalmers, King, Spencer, Wright, & Carter, 2005) to visually assess and score a participant’s oral hygiene. The OHAT used for this study was adapted from the version originally developed by Chalmers et al. (2005).

The OHAT was adapted based on literature, findings from the pilot study and the inputs of a dentist, to refine the criteria assessed, thus providing more meaningful data related to the current study as well as to the South African setting. A pilot study was conducted over a 4 week period, at the same research hospital, to evaluate the reliability and validity of the data collection instruments, and to improve upon the study design and instruments. Results of the pilot identified that the original version of the OHAT focused on providing a specific score for each patient based on the health of the oral cavity. However, to meet the objectives of the current study, more emphasis had to be placed on identifying and describing oral hygiene problems, rather than simply screening and scoring. Thus, the OHAT used for the purpose of this study was modified in the following ways. Whilst the eight categories of the original tool were maintained, more emphasis was placed on identifying and describing specific issues rather than simply scoring the responses. This allowed for additional structured observations that were added under each category to give a richer description of each area assessed and the scoring system was altered from three possible scores (0: healthy; 1: some changes; 2: unhealthy) to a range of 0–5. The altered scoring represented additional oral health abnormalities observed according to severity under each sub-category, which enabled the responses to not only indicate severity but also provide a descriptive result of the oral hygiene status.

#### Microbiology laboratory testing

The oral hygienist collected samples from the buccal site of the participants’ oral cavities, using cotton-tip swabs. This was a once-off collection taken after the completion of the CSE. The samples were transported to a biomedical-testing laboratory for microbiology laboratory, oral culture analysis.

## Data analysis

The clinical data from the MASA, the OHAT and the microbiology swab results were analysed, using descriptive statistics, namely univariate analysis, to describe and summarise patterns found in the data. Statistical analyses of data included frequency distribution, central tendency (mean, median and mode) and measures of variability (range and standard deviation).

Pulse oximetry and CA were used as adjuncts to the MASA assessment during the CSE, as a means to increase sensitivity and specificity of the MASA findings. To determine whether the relationship between the variables was statistically significant, categorical data from the PO, CA and MASA scores were tested using a test of independence, namely the Fischer’s exact test. The statistical analysis was performed using SPSS^TM^ Version 24, a *p*-value of ≤ 0.005 being considered significant. A cross-tabulation between (1) MASA scores and PO and (2) MASA scores and CA was done. More than 20% of the cells have an expected count of less than 5 and thus Fischer’s exact test was used. The MASA scores were split into three groups: (1) lower – score less than MASA quartile 1 value for the 40 respondents; (2) average – score between MASA quartile 1 and quartile 3 values; and (3) higher – score greater than MASA quartile 3. A significant correlation was found between PO and the MASA score. The *p* value was less than 0.005. Those who pass the PO have significantly higher MASA scores (almost double) than those who fail; thus confirming results of MASA. Similarly, the *p* value for CA to MASA scores was 0.000 indicating a significant association between the two. Results thus indicate that the two supplemental procedures PO and CA had a high rate of agreement with the MASA findings.

### Ethical considerations

The Biomedical Research Ethics Committee (BREC) of the University of KwaZulu-Natal, South Africa, approved this study (ethics reference number: BE454/15). The process of informed consent was specifically designed for the purpose of this study given that participants had co-morbid cognitive–linguistic disorders, which affected attention, memory, language and in turn communication abilities. To respect the rights and ensure the autonomy of participants who were unable to verbalise, Augmentative and Alternative Communication (AAC) charts were used to indicate yes or no, which was accepted as a form of consent. Furthermore, in cases where the participant was cognitively intact but unable to write or provide a signature, a thumbprint in the presence of a witness was considered as written consent. Where patients were unconscious or presented with severe language deficits, their family members and/or next of kin were approached to provide consent. Only participants for whom consent was obtained, were included in the study.

## Results

### Clinical features of dysphagia

Forty participants were initially identified as having dysphagia during sample selection. Out of the initial 40 participants recruited, 57.5% male (*n* = 23) and 42.5% female (*n* = 17), were confirmed to present with dysphagia in varying degrees of severity. Seventeen (42.5%) presented with severe, seven (17.5%) with moderate and four (10%) with mild dysphagia, as confirmed by the CSE. Twelve (30%) participants presented with little evidence of dysphagia (LED). As the study aimed to investigate oral hygiene issues present in patients with dysphagia specifically, the decision was made to include participants who presented with a MASA score of ≥ 178 indicating little evidence of disease (LED), as these patients still presented with lower scores in the oral preparatory phase (lip seal, tongue strength and coordination) and oral phases (bolus clearance, oral transit time) of the swallow. From this sample of participants who had confirmed dysphagia, the majority (70%) presented with a degree of risk for aspiration, ranging from a definite to possible risk.

### Clinical signs of dysphagia

According to the results of the CSE, 75% of participants presented with clinical signs of impaired efficacy of the swallow and 52.5% presented with clinical signs of impaired safety of the swallow. The five main clinical signs and symptoms of impaired efficacy of the swallow, as presented in Table 2, were poor lip seal (77.5%), reduced tongue strength (67.5%), poor tongue coordination (62.5%), impaired oral transit (90%) and ineffective bolus clearance (92.5%).

**TABLE 2:** Predominant deficits affecting efficacy of the swallow (*N* = 40).

Oral phase deficits	Variables	Number	%
1. Poor lip seal	Unable to assess	4	10.0
	No closure	4	10.0
	Incomplete seal	8	20.0
	Unilaterally weak and/or poor maintenance	9	22.5
	Mild impairment	6	15.0
	**Total**	**31**	**77.5**
	NAD	9	22.5
2. Reduced tongue strength	Gross weakness	20	50.0
	Unilateral weakness	7	17.5
	Minimal weakness	13	32.5
3. Poor tongue coordination	No movement	10	25.0
	Gross incoordination	4	10.0
	Mild incoordination	11	27.5
	**Total**	**25**	**62.5**
	NAD	15	37.5
4. Impaired oral transit	No movement observed	5	12.5
	Delay > 10 sec	6	15.0
	Delay > 5 sec	15	37.5
	Delay > 1 sec	10	2.0
	**Total**	**36**	**90.0**
	NAD	4	10.0
5. Ineffective bolus clearance from oral cavity	No clearance	8	20.0
	Some clearance and/or residue	13	32.5
	Significant clearance and/or minimal residue	16	40.0
	**Total**	**37**	**92.5**
	Fully cleared (NAD)	3	7.5

NAD, no abnormalities detected.

The main signs of impaired safety of the swallow (impaired cough, voice change, impaired hyolaryngeal elevation) are presented in Table 3. These signs were accompanied by oxygen desaturation and abnormal swallow sounds, with 52.5% of the participants demonstrating some signs of penetration and/or aspiration whilst swallowing. Signs of penetration were subjectively documented as gurgling during the swallow and coughing before, during or after swallowing which was detected in 12 (30%) and 9 (22.5%) participants respectively. Twenty-six (65%) participants demonstrated difficulties with hyolaryngeal elevation.

**TABLE 3:** Predominant deficits affecting the safety of the swallow (*N* = 40).

Pharyngeal phase deficits	Variables	Number	%
1. Impaired reflexive and voluntary cough	No reflexive cough observed	8	20.0
	Weak reflexive cough	15	37.5
	No attempt at voluntary cough	12	30.0
	Voluntary cough inadequate	8	20.0
	Voluntary cough bovine	8	20.0
	NAD	12	-
2. Change in voicing	Aphonic or unable to assess	10	25.0
	Wet and/or gurgly	9	22.5
	Hoarse	4	10.0
	Mild impairment	7	17.5
	**Total**	**30**	**75.0**
	NAD Reflexive	17	42.5
	NAD Voluntary	12	30.0
3. Impaired hyolaryngeal excursion	No swallow	1	2.5
	Pooling and/or gurgling (laryngeal elevation incomplete)	12	30.0
	Laryngeal elevation mildly Restricted or slow or incomplete	13	32.5
	**Total**	**26**	**65.0**
	NAD	14	35.0
4. Ineffective bolus clearance from oropharynx	No clearance	8	20.0
	Some clearance or residue	13	32.5
	Significant clearance or minimal residue	16	40.0
	**Total**	**37**	**92.5**
	Fully cleared	3	7.5

NAD, no abnormalities detected.

The reflexive and voluntary cough response was ineffective in 57.5% of the participants, and 70% could either not cough on command, or presented with an inadequate or non-explosive (bovine) cough attempt.

### Oral hygiene and degree of oral bacterial colonisation

Oral hygiene status was found to be very poor amongst all participants, with a high prevalence of bacterial colonisation, each participant presented with at least a minimum of one oral hygiene issue at the time of assessment, with a mean of 4.25, a median of 4 and a maximum of 7.

### Oral hygiene status

Table 4 presents the oral hygiene issues participants presented with. The most prevalent oral hygiene issues were related to the use of dentures, saliva, oral cleanliness and the tongue, with 80% having hygiene-related issues for the latter. The next most prevalent issue was related to the condition of the gums and tissues, with 52.5% of participants demonstrating issues such as dryness (15%), red or white patches (5%), signs of inflammation (15%), oral mucosal lesions (15%) and signs of possible infection (2%). Twenty-four participants (60%) had their natural dentition, which was in a good condition. Of the 25 who had dentures, a large majority (71.4%) experienced problems relating to their use, such as poor fit attributed to the dentures being loose (21.4%), or resulting in pressure or broken areas (7.2%). The presence of tartar build-up on dentures was identified in three (10.7%) participants.

**TABLE 4:** Oral hygiene indicators relating to the most prevalent conditions of the oral cavity.

Variable	Status	Oral hygiene indicators	Number	%
1. Gums and tissues	Normal	-	19	47.5
	Abnormal 21 (52.5%)	Dry and/or shiny	6	15.0
		Red and/or white	2	5.0
		Signs of inflammation	6	15.0
		Oral mucosal lesions	6	15.0
		Signs of possible fungal infection	1	2.0
2. Tongue	Normal	-	8	20.0
	Abnormal 32 (80%)	Some white patches	15	37.5
		Red	0	0.0
		Ulcerated and/or swollen	3	7.5
		Abnormal coating	13	32.5
		Unable to view	1	2.5
3. Oral cleanliness	Normal	-	7	17.5
	Abnormal 33 (82.5%)	Localised plaque	6	15.0
		Generalised plaque	7	17.5
		Tartar and/or calculus on teeth	3	7.5
		Accumulated saliva	4	10.0
		Food particles	13	32.5
4. Saliva	Normal Abnormal 24 (60%)	-	16	40.0
		Pooling of saliva	6	15.0
		Dry, sticky tissues	11	27.5
		Tissues are parched and red	3	7.5
		Sticky and/or ropey	3	7.5
		Discolouration of secretions	1	2.5

### Degree of oral bacterial colonisation

Of the 40 participants, only 15 (37.5%) presented with normal oral flora; thus, the majority (62.5%) presented with bacterial organisms that are not part of the resident oral flora. Of the 15 patients who presented with normal oral flora, 66.6% (*n* = 8) presented with LED according to their MASA scores. Higher MASA scores, 178 and above, were thus associated with normal oral flora. On the other hand, lower MASA scores, indicating a higher severity of dysphagia, were associated with an increased likelihood for the presence of pathogenic oral bacteria.

Seventeen (42.5%) had at least one bacteria strain present; seven (17.5%) had up to two strains and one (2.5%) participant had up to three bacterial strains present. Table 5, lists the most commonly occurring organisms isolated from the oral cavity of participants, these include (1) *Candida* and various strains of the *Candida* species (47.5%) and (2) opportunistic respiratory pathogens (37.5%). Twenty-two (47.5%) participants had only yeast; the remainder of the participants’ cultures had single isolates of opportunistic respiratory pathogens or opportunistic respiratory pathogens co-occurring with yeast.

**TABLE 5:** Bacterial organisms isolated from the oral cavities of participants and the degree of colonisation.

Organism isolated	Degree of colonisation							
	Normal		Scanty		Moderate		Profuse	
	*n*	%	*n*	%	*n*	%	*n*	%
***Candida***								
*Candida*species growth	0.0	0.0	0.0	0.0	1.0	2.5	0.0	0.0
*Candida albicans*growth	0.0	0.0	1.0	2.5	8.0	20.0	5.0	12.5
*Candida tropicalis*growth	0.0	0.0	0.0	0.0	1.0	2.5	0.0	0.0
*Candida glabrata* growth	0.0	0.0	0.0	0.0	1.0	2.5	2.0	5.0
**Gram-negative bacteria**								
*Staphylococcus aureus* growth	0.0	0.0	0.0	0.0	1.0	2.5	2.0	5.0
*Klebsiella pneumoniae* growth	0.0	0.0	0.0	0.0	0.0	0.0	4.0	10.0
*Enterococcus faecalis*	0.0	0.0	0.0	0.0	0.0	0.0	3.0	7.5
*Pseudomonas aeruginosa* growth	0.0	0.0	0.0	0.0	0.0	0.0	1.0	2.5
Group B *Streptococci* growth	0.0	0.0	1.0	2.5	0.0	0.0	1.0	2.5
*Stenotrophomonas maltophilia* growth	0.0	0.0	0.0	0.0	0.0	0.0	2.0	5.0
Normal oral flora	15.0	37.5	0.0	0.0	0.0	0.0	0.0	0.0

## Discussion

This study found that the majority of participants did not present with satisfactory oral hygiene, with a high prevalence of bacterial colonisation. Furthermore, poor oral hygiene status was associated with hospitalisation and not limited to those presenting with dysphagia. The findings support those of previous studies, which report the prevalence of oral health problems amongst acutely hospitalised patients to be very high (Danckert et al., 2016; Matthews et al., 2012; Tada & Hanada, 2010). In recent years, the interaction between opportunistic respiratory pathogens and *Candida albicans* has been reported (Dahlén, 2009). This finding was corroborated in the current study, as the most commonly occurring bacteria in the oral cavity of participants were (1) *Candida* species and (2) opportunistic respiratory pathogens. In five (17.5%) participants, *Candida albicans* was found to co-occur with an opportunistic respiratory pathogen.

In the context of oral health, stroke can cause hemiparesis and hemiplegia to the muscles of the pharynx, tongue and palate and the muscles of mastication, resulting in an impaired ability to chew and grind food and reduced oral clearance. Plaque biofilms are dislodged by the movement of the oral musculature, such as the cheeks and tongue, during speech and mastication (Kilian et al., 2016). Furthermore, studies have shown that effective salivary flow and swallowing is needed to clear bacteria-laden secretions from the oral and pharyngeal cavities (Ortega et al., 2015; Palmer, 2008). In this study, poor mechanical clearance of the bolus, which included the clearance of accumulated saliva, was found to be an area of significant difficulty for most participants with dysphagia. This resulted in oropharyngeal residue that was particularly related to tongue weakness and difficulties with coordination. According to Palmer et al. (2001), the reduction in mechanical clearance of potential pulmonary and oropharyngeal pathogens may be the first step in the path that leads to oral pharyngeal colonisation and pneumonia. Oropharyngeal residue that is not cleared promotes bacterial growth and invasion as pathogens have increased time in the mouth for proliferation. Reduction in mechanical clearance could also account for the finding that all participants presented with deficient oral cleanliness related to the accumulation of plaque, saliva and debris. The motor function of the tongue is described to be related to its self-cleaning function. Reduced activity as a result of weakness possibly accounts for the finding that 80% of the study participants had oral hygiene issues related to the tongue, more specific to tongue coating. This finding is concerning as studies have identified the dorsum of the tongue as a major oral site of bacterial multiplication, as well as a major source for the bacteria found in saliva. This indicates that many salivary bacteria mirrors those coming from the flora of the dorsum of the tongue (Danser, Go´mez, & Van der Weijden, 2003; Kikutani et al., 2009; Pace & McCullough, 2010) which could account for the high prevalence of pathogenic bacteria found in the oral cavities of participants with dysphagia. Participants with dysphagia were found to have more food debris, plaque, poorer oral clearance and more denture problems. This can be ascribed to functional impairments of the oral phase of the swallow, such as reduced orofacial muscular control and loss of oral sensory function, which are symptoms and complications of disease processes, such as cerebrovascular accident (CVA) that affect the efficacy of the swallow. These deficits may thus have the potential to significantly affect oral hygiene, creating a favourable environment for pathogenic bacteria to grow and colonise.

It has been extensively researched and is well known that oral microbiota contributes to oral and general well-being and its loss can be detrimental to the health of the individual. Numerous studies have implicated oral colonisation with pathogenic bacteria as a precursor condition contributing to AP risk. Participants in the current study presented with an increased number of pathogenic oral bacteria, with only 15 participants presenting with normal oral flora. It was interesting to note that the larger majority of participants presented with bacteria strains commonly associated with the acquisition of nosocomial infections, such as bacterial pneumonia. Infection control efforts are applied in hospitals to prevent nosocomial infections, with microbiology surveillance often put into place to detect harmful microorganisms before they have the chance to ensue disease. Research states that as long as the microbial load is not too high, the harmony between pathogenic bacteria and oral resident flora will not be tilted toward infection (Danser et al., 2003; Rautemaa et al, 2007). However, the results of this study indicate that when bacterial species were found in the oral cavities of participants, the virulence was already quite high. This bacterial virulence suggested the delicate oral homeostasis had potentially already been tipped toward infection and if not treated could potentially lead to systemic disease such as AP. In this study, 70% of participants presented with some degree of risk for aspiration -– thus presenting with an increased likelihood to aspirate pathogenic bacteria, which could overburden the host defence mechanisms and lead to infection, resulting in longer hospital stays, costly care and possible death (Terpenning & Shay, 2002). The monitoring of oral bacterial colonisation may thus be crucial in determining the risk for pneumonia in hospitalised patients who present with risk factors such as dysphagia (Yoon & Steele, 2013).

Pain is a key indicator for patients and medical professionals, such as nurses, that something is wrong (Germossa, Hellesø, & Sjetne, 2019). An interesting finding of the study was that although oral hygiene was poor, 83% of participants (*n* = 33) demonstrated no signs of oral and/or dental pain, with no participants having any verbal reports of pain. Because of the nature of the clinical population, such as increased reliance on others for care including oral hygiene, communication disorders and the resulting inability to effectively communicate their needs, people with dysphagia can be regarded as a voiceless population. Furthermore, it is known that oral hygiene issues may not be painful and often remain asymptomatic (Dahlén, 2009), requiring further inspection. These patient factors in combination with the asymptomatic nature of oral hygiene problems highlight the silent nature of oral diseases.

Thus, because of the nature of the patient population, interprofessional collaboration and problem solving between dental staff, nurses and speech-language therapists is required to address patient oral care during hospitalisation.

## Conclusion

This study demonstrated that the oral hygiene status of hospitalised patients who present with dysphagia is concerning. Patients with dysphagia may present with many oral hygiene problems, which may lead to a high prevalence of colonisation of respiratory pathogens. The findings of this study not only corroborate the findings from previous research, which indicated that oral hygiene is poor during hospitalisation, but also provide novel information regarding the oral hygiene problems people with dysphagia present with. Furthermore, it highlights the fact that oropharyngeal swallowing disorders in conjunction with poor oral hygiene, constitute an increasing problem for the often silent hospitalised patient who presents with co-morbid communication disorders. Hospitalised patients with dysphagia thus require greater vigilance in oral care, which may include improved screening of oral hygiene and frequent and professional dental care during hospitalisation, to minimise the potential effect of this undetected, silent killer.

## Limitations of the study

The study employed a relatively small sample size of only 40 participants. Furthermore, the study location was a sub-acute rehabilitation hospital based within the private sector in South Africa. Patients admitted there thus had private medical aids and would present with very different socio-economic characteristics compared to those admitted to a government facility. As a result, the researcher does not expect the sample from this study to be representative of all hospitalised patients in South Africa. Future research in South Africa should focus on obtaining population representative data by investigating oral hygiene in both private and government institutions, as well as utilising a larger sample size.
